# Spatiotemporal Transition in the Role of Synaptic Inhibition to the Tail Beat Rhythm of Developing Larval Zebrafish

**DOI:** 10.1523/ENEURO.0508-18.2020

**Published:** 2020-02-13

**Authors:** Yann Roussel, Melissa Paradis, Stephanie F. Gaudreau, Ben W. Lindsey, Tuan V. Bui

**Affiliations:** 1 Brain and Mind Research Institute, Centre for Neural Dynamics, Department of Biology, University of Ottawa, Ottawa K1N 6N5, Canada; 2 Rady Faculty of Health Sciences, Max Rady College of Medicine, Department of Human Anatomy and Cell Science, University of Manitoba, Winnipeg R3E 0J9, Canada

**Keywords:** motor maturation, network oscillators, spinal locomotor circuits, swimming, synaptic inhibition, zebrafish

## Abstract

Significant maturation of swimming in zebrafish (*Danio rerio*) occurs within the first few days of life when fish transition from coiling movements to burst swimming and then to beat-and-glide swimming. This maturation occurs against a backdrop of numerous developmental changes - neurogenesis, a transition from predominantly electrical to chemical-based neurotransmission, and refinement of intrinsic properties. There is evidence that spinal locomotor circuits undergo fundamental changes as the zebrafish transitions from burst to beat-and-glide swimming. Our electrophysiological recordings confirm that the operation of spinal locomotor circuits becomes increasingly reliant on glycinergic neurotransmission for rhythmogenesis governing the rhythm of tail beats. This transition occurred at the same time that we observed a change in rhythmicity of synaptic inhibition to spinal motoneurons (MNs). When we examined whether the transition from weakly to strongly glycinergic dependent rhythmogenesis occurred at a uniform pace across the length of the spinal cord, we found that this transition occurred earlier at caudal segments than at rostral segments of the spinal cord. Furthermore, while this rhythmogenic transition occurred when fish transition from burst swimming to beat-and-glide swimming, these two transitions were not interdependent. These results suggest that there is a developmental transition in the operation of spinal locomotor circuits that is gradually set in place in the spinal cord in a caudo-rostral temporal sequence.

## Visual Abstract

**Figure:**
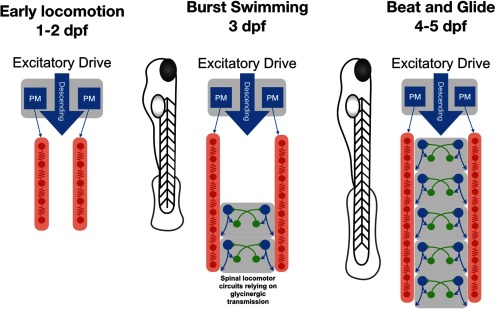

## Significance Statement

The neural circuits controlling movements must adapt to the development of the body to enable more refined and complex movements. Our results confirm a time-window in which the mechanisms for generating the rhythm driving tail beats in developing zebrafish shift from weakly relying on synaptic inhibition to becoming strongly dependent on synaptic inhibition. Surprisingly, this transition occurs at different developmental time points along the length of the spinal cord, in a direction opposite to the direction of growth of the body. Our findings emphasize that the maturation of motor control by the nervous system results from fundamental changes in the operation of the spinal circuits that underlie these movements and these changes occur at different times across the nervous system.

## Introduction

Motor control by the nervous system gradually matures during development as organisms acquire new movements and refine existing ones. This maturation of motor control occurs at a time when the nervous system is still developing; neurogenesis is still ongoing, new neural circuits are forming, and intrinsic properties are being modified (Favero et al., 2014) to support novel and refined network function.

Zebrafish exhibit a rapid series of changes in swimming movements as they develop. Large single tail bends (coils) are first seen at 1 d postfertilization (dpf), followed by a transition to multiple coils towards the end of the first day of development. Coiling movements are then replaced by swimming movements. These are first seen as long (a few seconds in duration) but infrequent episodes of smaller tail bends that emerge at 2–3 dpf (burst swimming). By 4–5 dpf, burst swimming is replaced by more frequent trains of shorter (hundreds of milliseconds) swimming episodes regularly interspersed with silent “gliding periods” (beat-and-glide swimming; Drapeau et al., 2002). Mounting evidence suggests that there are a number of changes in spinal locomotor circuits as developing zebrafish transition between these different modes of locomotion.

Early embryonic locomotor activity is generated by spinal circuits interconnected by electrical synapses (Saint-Amant and Drapeau, 2001) that are putatively driven rhythmically by a small kernel of rostrally-located pacemakers, the previously described ipsilateral caudal (IC) spinal interneurons (Tong and McDearmid, 2012). IC neurons display endogenous bursting via persistent sodium currents. Whether an electrical network driven by a small pacemaker kernel is sufficient to accommodate later more complex locomotor behaviors produced by the growing body of the developing zebrafish (Kimmel et al., 1995) is not clear. While gap junctions may easily generate rapid, synchronous activation of motoneurons (MNs) to facilitate coiling, a transition towards chemical synapses would facilitate a more refined spatiotemporal control of a larger spinal network (Bernhardt et al., 1990) for sustained repeated tail beats. Indeed, the electrical network in the zebrafish spinal cord has been shown to quickly evolve into a hybrid electrochemical network involving both electrical and chemical synapses capable of generating more complex movements (multiple-coiling; Knogler et al., 2014).

Observations that blocking glutamatergic transmission at 3–4 dpf perturbs the generation of tail beats (Buss and Drapeau, 2001) serve as further evidence for the emergence of chemical neurotransmission in the generation of tail beats. However, the role of synaptic inhibition to the generation of tail beats is not as clear. In the developing zebrafish spinal cord, the main inhibitory neurotransmitter is glycine (Buss and Drapeau, 2001). Application of strychnine, at 3 dpf leads to bilateral contractions, presumably due to disruption of commissural inhibition (Buss and Drapeau, 2001). However, the activity of MNs following application of strychnine remains rhythmic during spontaneous or light-induced swimming episodes (Buss and Drapeau, 2001). Conversely, strychnine can disturb the rhythm of tail beats in developing zebrafish during electrically-induced swimming at 4–5 dpf (McLean et al., 2008), or NMDA-induced swimming at 3 dpf (McDearmid and Drapeau, 2006), and at 4 dpf (Wiggin et al., 2014). Activation of NMDA receptors promotes oscillatory activity in neurons due to the voltage-dependence of the Mg^2+^ block of this channel (Schmidt et al., 1998). Therefore, en masse application of NMDA could produce rhythmic activity in spinal locomotor circuits that does not completely resemble endogenous rhythmic activity during locomotion. This is reinforced by studies showing differences in the characteristics of swimming activity, and in the membrane oscillations and action potentials of MNs during locomotor activity induced with or without NMDA (Talpalar and Kiehn, 2010; Kyriakatos et al., 2011). Thus, the exact contributions of synaptic excitation and inhibition to rhythmogenesis could be affected by the availability of this agonist to all spinal neurons. Therefore, we sought to investigate the role of glycinergic neurotransmission to the generation of the rhythm governing tail beats during spontaneous or light-induced swimming.

Our study confirms that synaptic inhibition is involved in the rhythm of tail beats of developing zebrafish between 3–5 dpf. In this time window, the rhythm transitions from a weakly glycine-dependent rhythm (WGDR) to a strongly glycine-dependent rhythm (SGDR). Furthermore, we found that this transition from a WGDR to an SGDR does not occur simultaneously across the length of the fish, but rather progresses in a caudo-rostral direction, occurring first in newer caudal segments of the developing fish. Our findings suggest that the role of synaptic inhibition to the generation of the rhythm of tail beats within each swim episode changes during the development of the larval zebrafish.

## Materials and Methods

### Animal care

We maintained adult wild-type and transgenic zebrafish according to standard procedures (Westerfield, 2000). All experiments were performed in accordance with the protocol approved by the University of Ottawa animal care committee (BL-2038). The light cycle in the zebrafish room was set as 14/10 h light/dark, with lights on at 9 A.M. and off at 11 P.M. Water temperature and water chemistry in the fish system were maintained in the following ranges: 29–30°C, pH 7.5–8.0, and conductivity 120–150 μS/cm. Zebrafish embryos whose sex are developmentally undetermined were collected immediately after breeding and transferred in 10 cm diameter plastic Petri dishes in E3 embryo medium. Each Petri dish had a maximum density of 50 embryos in 10-cm diameter plastic Petri dishes. We placed Petri dishes in an incubator maintained at 28.5°C in E3 embryo media. Transgenic embryos were screened at 24–48 h postfertilization (hpf) using a fluorescent dissecting microscope to identify positive embryos based on fluorescent reporter expression.

### Video recording and analysis

The heads of 4 dpf larvae were embedded in 1.2% agarose gel allowing the rest of the body to be freely moving. High-speed (500 fps) video recordings of spontaneous swimming were conducted with a Basler acA640-750 μm camera using infrared light. Frame by frame analysis was performed using ImageJ (RRID:SCR_003070) embedded functions. We developed a custom macro based on previous work from Fontaine et al. (2008) as follows: A region of interest (ROI) was drawn to span the area bound by the pectoral fins and the tail. Then, a threshold was applied to convert the frame to a binary picture (black and white). Each pixel was extended to smooth the edge of the body of the fish. Any blank space within the reconstructed fish was filled in. Next, the ImageJ “skeletonize” function was executed to extract the midline of the body, and *x-y* coordinates of the midline were extracted. We then transferred the *x-y* coordinates into a custom Python program that segmented the body in 30 segments from the caudal to the rostral end. The position of the body was normalized so that the extreme caudal segment was 0.0 and the extreme rostral segment was 1.0. For every frame, the program computed the angles between each segment using the arctangent of the derivative and compared this value to the rest position (given the relatively small angles of body curvature for swimming episodes, there was no problem related to the arctangent definition). Finally, a Fourier transform of each body segment was performed using the fast Fourier transform (FFT) algorithm from the numpy Python library (RRID:SCR_008633).

### Animal preparation for electrophysiology

Three- to 5-dpf wild-type and transgenic larvae were anesthetized in 0.02% tricaine solution and pinned down through the notochord in a Sylgard (Dow Corning) coated dish, one pin above the swim bladder and a second caudal to the anus. We made pins from 0.01 mm tungsten wires. A skin flap was created with fine forceps near one of the pins and used to remove the rest of the skin between the two pins. The fish was then bathed in aCSF (134 mM NaCl, 2.9 mM KCl, 1.2 mM MgCl_2_, 2.1 mM CaCl_2_, 10 mM dextrose, and 10 mM HEPES) containing 1 mg/ml collagenase (Millipore Sigma) solution for 20 min. Muscles were removed over five to nine somites by applying suction through a 15-μm glass micropipette.

### Extracellular recordings

We backfilled 10-μm borosilicate glass microelectrodes (outer diameter: 1.5 mm; inner diameter: 0.86 mm; A-M. Systems; catalog #596800) with 2 M NaCl solution (Baraban, 2013). We approached the spinal cord targeting a zone just dorsal to a ventral root, and a light suction was applied to seal the ventral root and a portion of the spinal cord. In a subset of experiments, we applied a second microelectrode to a motor nerve at a somite adjacent to the recorded ventral root. Electrical activity was recorded in current clamp mode, amplified and filtered at 1 kHz with a Multiclamp 700B from Axon Instruments (Molecular Devices) and finally digitized with a Digidata 1550 (Molecular Devices) to be stored on a computer. 50/60 Hz noise was attenuated using a Hum Bug Noise Eliminator (Quest Scientific). Light pulses were occasionally applied to increase the occurrence of swimming events. Both spontaneous and light-evoked swimming events were pooled together since no significant difference in duration and tail beat frequency was observed (Buss and Drapeau, 2001). Strychnine (4 μM; Millipore Sigma) or bicuculline (10 μM; Millipore Sigma) were made in aCSF and applied to the bath to test the effects of blocking glycinergic or GABAergic transmission, respectively, on rhythmic tail beats. In a subset of experiments, fictive swimming was induced in spinalized larvae by applying NMDA to the bath (1–1.5 mm). In all experiments, somites were numbered from 1 to 9, somite 1 being the sixth somite rostral to the anus and somite 9 being the third somite caudal to the anus.

### Intracellular recordings

Intracellular recordings of MNs were made in wild-type fish. After muscle removal, we backfilled borosilicate glass microelectrodes with intracellular recording solution (16 mM KCl, 116 mM K-gluconate, 4 mM MgCl_2_, 10 mM HEPES, and 10 mM EGTA) containing 0.1% sulforhodamine B (Millipore Sigma). Sulforhodamine was added to the intracellular recording solution to visualize cell morphology, in particular, axonal morphology to identify MNs. Based on relative dorsoventral location, targeted MNs were approached while applying slight positive pressure. Prior to entering the spinal cord, the electrode was briefly stopped to break the dura with positive pressure only. We next introduced the electrode into the spinal cord, and we decreased the positive pressure to finalize the approach. After ensuring that no tissue was present between the micropipette tip and the targeted MN, the pressure was released allowing the formation of a GΩ seal in most cases. If required, we applied a very light suction to form a seal. A holding potential of −65 mV was then applied, and after capacitance compensation, the seal was broken with a series of light suction pulses. We compensated the series resistance during recordings (70–80%). MNs were identified by axonal projections that were directed towards putative ventral roots through visual inspection of cell morphology using sulforhodamine B labeling loaded during the intracellular recordings.

### Transgenic lines

Two transgenic green fluorescence protein (GFP)-reporter lines were used to identify changes in spinal neuron populations along the rostral-caudal axis: Tg(*isl1:GFP*) and Tg(*chx10:GFP*). Specifically, the *isl1* line was used to visualize secondary MNs (sMNs), whereas the *chx10* line marked V2a (CiD) interneurons (Ampatzis et al., 2014; Ljunggren et al., 2014; Stil and Drapeau, 2016). Additionally, the metronidazole (MTZ) inducible transgenic line, Tg(*dat:CFP:NTR*) (Godoy et al., 2015), was employed to eliminate dopamine-expressing neurons, with activation of this system as described below. The Tg(*dat:CFP-NTR*) line was kindly provided by the lab of Marc Ekker (Godoy et al., 2015; ZDB-TGCONSTRCT-160128–1).

### Chemogenetic ablation of DA neurons

We followed the same protocol described by Godoy and colleagues (Godoy et al., 2015). Briefly, homozygous Tg(*dat*:*CFP-NTR*) embryos were dechorionated manually. We treated embryos with 5 mM MTZ (Millipore Sigma) dissolved in 0.1% dimethylsulfoxide in E3 embryo medium (Westerfield, 2000) from 1 to 2 dpf, then increased the concentration of MTZ to 7.5 mM. MTZ solution was replaced every day until larvae reached 4 dpf. Embryos were kept at 28.5°C in the dark in 10-cm dishes with one fish per milliliter of solution. At 4 dpf, fish were rinsed in E3 media thrice before being transferred to a clean dish with fresh embryo media for an additional day, with experiments performed over this time.

### Tissue processing for immunohistochemistry

Larvae were sacrificed in a solution of 0.4% MS-222 (i.e., tricaine), and immediately fixed in 4% paraformaldehyde (PFA; Sigma; 158127) diluted in phosphate buffer (pH 7.4) for 1 h at room temperature in the dark. Whole animals were next rinsed in buffer before being cryo-embedded in a mixture of fish gelatin (Sigma; G7041) and sucrose (VWR; VWRC0335) in PBS. Larval brain and spinal cords were then sectioned in sagittal planes using a Leica CS3080 Cryostat at a thickness of 20 μm and stored at −80°C until staining.

### Immunohistochemistry

Sheep anti-GFP primary antibody (Bio-Rad/AbD Serotec catalog #4745–1051, RRID:AB_619712; 1:500) was used to amplify the GFP signal for cell counting in the spinal cord of 3-dpf Tg(*isl1*:*GFP*) zebrafish. Mouse anti-zn-8 primary antibody (Zebrafish International Resource Center catalog #zn-8, RRID:AB_10013774; 1:300) was used to label most sMNs and is described as being identical to the zn-5 antibody staining pattern previously documented (Menelaou and Svoboda, 2009). To visualize CFP-positive dopaminergic cells in Tg(*dat*:*CFP-NTR*) larvae, a monoclonal mouse primary antibody against CFP was used (Clontech Laboratories, Inc. catalog #632380, RRID:AB_10013427; 1:400). Primary antibodies were conjugated to donkey anti-sheep Alexa Fluor 488 (Thermo Fisher Scientific, catalog #A-11 015, RRID:AB_2534082; 1:500) or goat anti-mouse Alexa Fluor 555 (Thermo Fisher Scientific, catalog #A-21 425, RRID:AB_2535846; 1:500) secondary antibodies.

In brief, immunohistochemistry was performed by rehydrating sections in 1× PBS with 0.3% Triton X-100 before primary antibody staining overnight at 4°C. The next day, tissue was rinsed with buffer before being incubated in the appropriate secondary antibody for 1 h at room temperature in the dark. All antibodies were diluted in 1× PBS buffer above, with 4′,6-diamidino-2-phenylindole (DAPI) counterstain included during secondary incubation to visualize cell nuclei. Thereafter, sections were rinsed to reduce non-specific binding and mounted using Immuno-Mount (Thermo Scientific) for imaging.

### Imaging and cell counts

Spinal neurons were counted in Tg(*isl1*:*GFP*) and Tg(*chx10*:*GFP*) 3-dpf larvae. For wholemount imaging of Tg(*chx10:GFP*) larvae, animals were anesthetized in 0.02% tricaine and pinned laterally through the notochord in a Sylgard coated dish. Imaging was completed using a Zeiss Examiner.D1 microscope equipped with an Axiocam 506 mono Zeiss camera and an X-Cite 120 LEDmini (Excelitas Technologies) light excitation source for fluorescence; 100-μm deep Z-stacks were performed using Zen pro software, and we performed cell counts manually.

Immunostained spinal cord sections of Tg(*isl1*:*GFP*) larvae and brain sections of Tg(*dat*:*CFP-NTR*) larvae were imaged using an Olympus FV1000 spectral LSM confocal microscope with Z-stack intervals of 0.5 μm. Imaging was performed at 20× and 40× magnifications, with a resolution of 1024 × 10^24^ pixels. We performed three to four independent counts for each Z-stack using Fluoview or ImageJ, and means were computed.

### Data analysis

#### Autocorrelation and Peak_20–40_ detection

Swimming episodes were characterized as long-duration (over 200 ms) bursts of high-frequency electrical activity. Tail beats were represented primarily by 20- to 40-Hz signaling (see Results). In some cases, most notably when strychnine was present at 4 and 5 dpf, swimming episodes were characterized instead as long-duration (over 200 ms) voltage changes with or without high-frequency signals superimposed. To quantify the effect of strychnine on rhythmic motor activity, and of different holding potentials on IPSCs, we developed a simple peak detection algorithm to detect the presence of a peak between 20 and 40 Hz in the autocorrelation function of each swimming episode trace (Peak_20–40_). Raw traces were first bandpass filtered between 1 and 200 Hz. We extracted ten 200-ms epochs during swimming episodes. Because the fictive swimming behavior matures from a few long episodes (several seconds) at 3 dpf to multiple shorter (few hundreds of milliseconds) episodes at 5 dpf, we chose 200-ms epochs from recordings at all ages to keep the analysis consistent. We computed autocorrelation functions for each epoch using Clampfit (Molecular Devices). Then we fit the autocorrelation function as a second-order polynomial function (in Clampfit):

$$Autocorr_{fit}(\tau )=a_{0}+a_{1}\cdot \tau +a_{2}\cdot \tau^{2}.$$

The fit was performed on the segment of the autocorrelation function between 20- and 50-ms time delay (*x*-axis of the autocorrelation function), which corresponds to a frequency range of 20–50 Hz. Finally, we applied the following conditions to the fitting parameters to ensure that the peak of the polynomial function was within the targeted 20- to 40-Hz range (first condition below), and that the polynomial function was concave down (second condition below):

$$25\;\text{ms}\;<\;\;\frac{-a_{1}}{{2a}_{2}}<\;50\;\text{ms}\;\text{and }\;\;a_{2}\;<0.$$

Only if all conditions were respected was a peak considered to be detected. Using the above conditions for all of our experiments gave us an average Peak_20–40_ detection rate above 80% under control conditions. Animals where the Peak_20–40_ score of recorded swimming episodes in the absence of any pharmacological interventions were smaller than 50% (meaning that a peak in the autocorrelation function was detected in the 25- to 50-ms time delay range less than half of the time), were considered as arrhythmic, and their recordings were discarded (29% rejection rate). The only exception to this rejection rule were recordings at somite 1 at 5 dpf, where two thirds of swimming episodes had Peak_20–40_ scores <50%. This was consistently observed for three separate experimenters. While the reasons for this higher rate of Peak_20–40_ scores at somite 1 at 5 dpf being below 50% is not clear, we chose to keep these recordings as part of our sample rather than having a greater rejection rate for this subset of the recordings.

#### Short-time Fourier transform

The frequency components of swimming under different conditions were computed using the short-time Fourier transform algorithm in Python; 30-s-long recordings were bandpass filtered between 1 and 200 Hz; then a Fourier transform was performed on every 375-ms epochs of the recording. Finally, we plotted all Fourier transforms as heat maps in Python.

#### Modelling

The reductive mathematical model consisted of two coupled oscillators coded in Python: a classical harmonic oscillator with a natural frequency $\omega_{0}$ of 20 Hz, driving a damped oscillator with the same natural frequency $\omega_{0}$ and a damping coefficient ζ. The damping oscillator receives an input from the harmonic oscillator characterized as: $F_{0}(t)=F_{0}sin(\omega_{0}t)$, with $F_{0}$ the coupling coefficient.

In addition, both oscillators receive a « go signal » from higher centers (i.e., a tonic excitation) $F_{1}(t)$ defined as a step function:

$$F_{1}(t)=\{\begin{array}{c} F_{1}\;if\;t_{1}\leq t\leq t_{2}\\ 0\;else \end{array}.$$

$F_{1}$ is a constant and $t_{1}$ and $t_{2}$ are the time interval limits during which the step signal is emitted. Thus, the harmonic oscillator (representing the pacemaker IC neurons) is only active during the time interval $\Delta t=t_{2}-t_{1}$. The damped oscillator (representing a half-center unit oscillator) receives the input $F_{0}(t)=F_{0}sin(\omega_{0}t)$ only for the same interval Δt. The output of the network, read at the output of the damped oscillator, is a solution of a simple ODE:

$$\frac{d^{2}x}{dt^{2}}+2\zeta \omega_{0}\frac{dx}{dt}+\omega_{0}^{2}=F_{0}sin(\omega_{0}t)+F_{1}(t),$$

where *x* is our oscillating variable from the damped oscillator (homologous to the membrane potential of MNs in our case). ζ represents the strength of reciprocal inhibition in a half-center rhythm generation mechanism and consequently, it represents the application or absence of strychnine. A high ζ models the application of strychnine preventing our half-center from oscillating. $F_{0}$ represents the coupling coefficient between the two oscillators. A high $F_{0}$ represents a strong coupling between the harmonic and the damped oscillator. A low $F_{0}$ represents a weak coupling, such as in the presence of a gap-junction blocker or due to the integration of new neurons into spinal half-center units that are not directly connected to the IC pacemaker neurons.

We solved the ODE in Python using different parameters: Strong coupling $F_{0}=0.5$, weak coupling $F_{0}=0.01$, strong reciprocal inhibition ζ=0.1 and weak reciprocal inhibition ζ=0.5. We finally computed and plotted the autocorrelation function from the output. The code for the model can be accessed at https://github.com/bui-lab/code.

### Experimental design and statistical analysis

We did not perform an explicit power analysis before the study. Statistical analysis was performed using Apple Numbers or Microsoft Excel. No animals or data were excluded except as indicated above to ensure proper swimming before addition of strychnine. A critical value of 0.05 was used to determine statistical significance. This critical value was corrected when performing multiple tests using the Bonferroni correction. While we provide exact *p* values from the Student’s *t* tests, asterisks are used in figures to denote statistical significance after the critical value has been adjusted using the Bonferroni correction. Paired Student’s *t* tests were used to analyze data collected in the same fish in different conditions (e.g., control vs strychnine) or locations (e.g., rostral vs caudal somites of the same fish). Unpaired Student’s *t* tests were used to analyze data collected in different fish (e.g., fish at different ages).

## Results

### The effect of strychnine on rhythmogenesis is strengthened from 3 to 5 dpf

**Figure 1.**
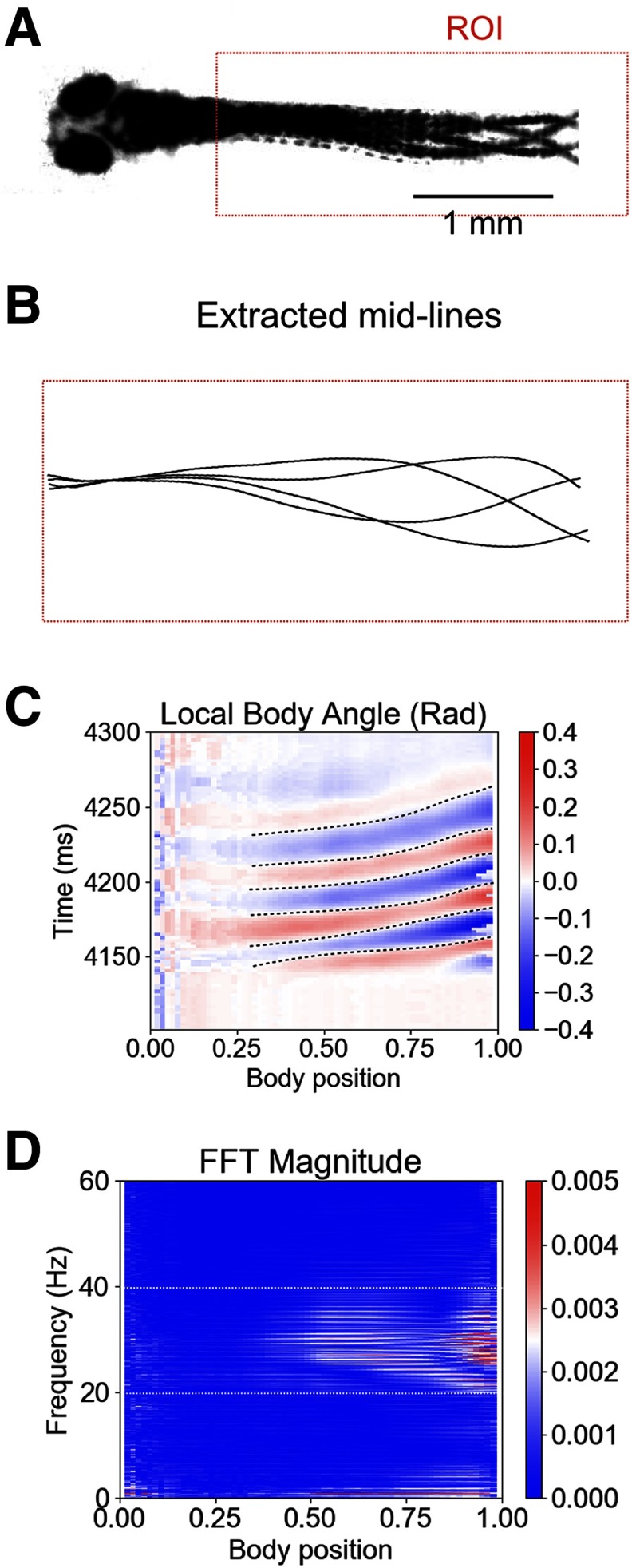
Larval zebrafish tail beat frequency during swimming is mainly between 20 and 40 Hz. ***A***, Four superposed frames recorded during a single swimming episode of a 4-dpf larval fish. The red dotted box is the ROI in which the frame by frame analysis was performed. ***B***, Examples of extracted midlines of the fish body over a swimming episode. ***C***, Heat map of the local body angle amplitude. The fish body was divided into thirty segments from the caudal to the rostral end. The position of the body has been normalized such that the extreme caudal segment is 1.0, and the extreme rostral segment is 0.0. For every frame, the angle of each segment was computed and compared with the resting position. The resulting amplitude was assigned a color, blue for negative angles and red for positive angles. Black dotted lines are used to illustrate successive tail beats. ***D***, For each body segment, a FFT was applied on a swimming episode and the result plotted as a heat map. Note that FFT outputs only positive values. The white dotted box highlights the 20- to 40-Hz range where the frequencies of tail beats reside.

To study the rhythmogenic mechanism underlying tail beats in 3- to 5-dpf larval zebrafish, we first identified the main frequency components of body bends during swimming activity recorded using high-speed video recordings made at 4 dpf (Fig. 1*A*). As reported previously (Muller, 2004; McLean et al., 2007; Fontaine et al., 2008), we observed that the maximum tail bend amplitudes were located very caudally (Fig. 1*B*,*C*) and that body oscillations were at a frequency between 20 and 40 Hz (Fig. 1*D*). Thus, we focused on this frequency range to conduct our analysis of the role of glycine to rhythmogenesis of tail beats for larval swimming.

**Figure 2.**
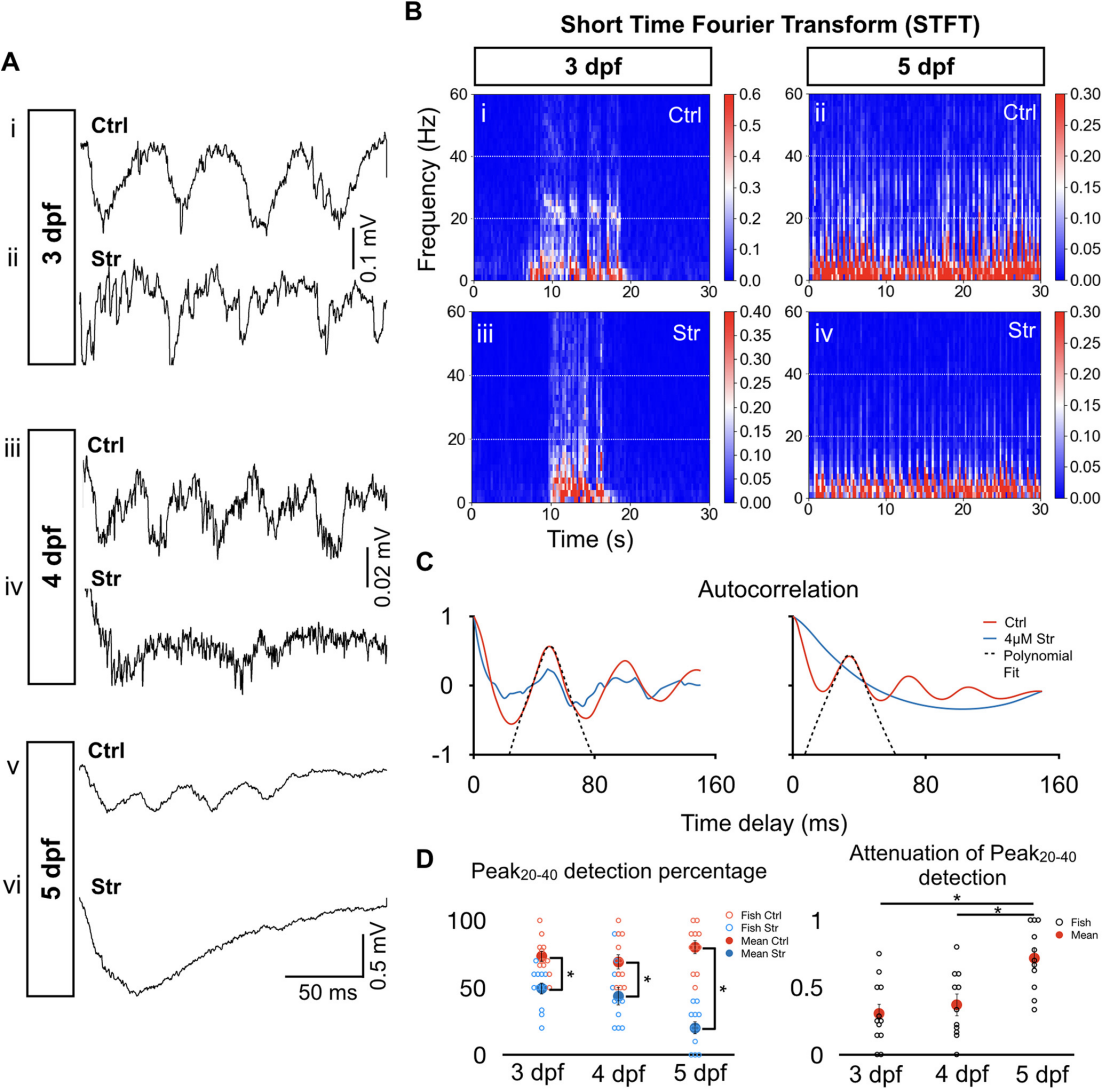
Greater effect of strychnine on tail beat rhythmicity at 5 dpf than at 3–4 dpf. ***A***, Typical extracellular recordings of tail beats during single swimming episodes under control (Ctrl) and 4 μM strychnine (Str) conditions at 3 dpf (***Ai***, ***Aii***), 4 dpf (***Aiii***, ***Aiv***), and 5 dpf (***Av***, ***Avi***). Arrowheads indicate examples of tail beats. Note the relative lack of tail beats at 4 and 5 dpf under strychnine condition (***Aiv***, ***Avi***). ***B***, Short-time Fourier transform (STFT) of a 30-s-long recording of spinal cord activity under control and 4 μM strychnine conditions at 3 dpf (***Bi***, ***Bii***) and 5 dpf (***Biii***, ***Biv***). White dotted lines mark the 20- to 40-Hz frequency range. We can see local maxima in the 20- to 40-Hz range for both 3-dpf plots and the control 5-dpf plot but not for the strychnine-positive 5-dpf plot. ***C***, Example of the autocorrelation function of typical 3- and 5-dpf traces (left and right panel, respectively) under control condition (blue) and strychnine application (red). A polynomial fit (dashed line displays fit to the control trace) was performed to the values of the autocorrelation function within the 20- to 50-ms time delay range. Peak detection was performed to detect the presence of a peak in the polynomial fit within the 25- to 50-ms time delay range, which corresponds to the 20- to 40-Hz frequency range (see Materials and Methods). ***D***, Left, Results from Peak_20–40_ detection algorithm for autocorrelation functions under control (blue) and strychnine (red) conditions. Data from somites 4–6 were pooled together. Right, Attenuation of Peak_20–40_ detection computed as 1 – (Peak_20–40_Strychnine/ Peak_20–40_Ctrl). *N* = 120 episodes (10 per fish, 12 fish) at 3 dpf, and *N* = 110 episodes (10 per fish, 11 fish) at each of 4 dpf and 5 dpf. Left, Two-tailed paired Student’s *t* test (at 3-dpf Ctrl-Str, *p* = 0.0014; at 4-dpf Ctrl-Str, *p* = 0.0029; and at 5-dpf Ctrl-Str, *p* = 3 × 10^−5^). Right, One-way ANOVA (*F*_(2,31)_ = 16.69, *p* = 1 × 10^−5^) followed by two-tailed unpaired Student’s *t* test (3–4 dpf, *p* = 0.5283; 3–5 dpf, *p* = 0.0005; and 4–5 dpf, *p* = 0.0032); *p < 0.0166, which indicates significance following Bonferroni’s multiple-comparisons correction. Open circles represent the scores of each individual. Solid circles are the averages for every age and condition. Top horizontal bars display result of tests between the two groups at each end of the bars. Error bars display SEM.

To study the effects of glycinergic neurotransmission to rhythmic tail beats between 3 and 5 dpf, we blocked glycinergic neurotransmission using 4 μM strychnine, a glycine antagonist, during spontaneous or light-induced swimming episodes. Typical control traces of ventral root recordings are shown in Figure 2 with spectral analysis of frequency components of recorded swimming episodes (Fig. 2*A*,*B*); 20- to 40-Hz oscillations can be observed in control recordings at 3, 4, and 5 dpf as confirmed by the spectral analysis. After strychnine application, oscillations in the 20- to 40-Hz range were attenuated at all ages tested (Fig. 2*A*,*B*). However, we could still see typical oscillations in more than half of the fictive swimming events at 3 dpf (Fig. 2*B*), consistent with previous observations using comparable concentrations of strychnine (6 μM) in an NMDA-induced spinalized preparation (McDearmid and Drapeau, 2006). The continued presence of oscillations was in contrast to 5 dpf when strychnine application ablated oscillations within the 20- to 40-Hz range (Fig. 2*Avi*), in line with earlier reports describing electrically-induced swimming (McLean et al., 2008). To quantify the effects of strychnine between 3 and 5 dpf, we plotted autocorrelation functions of 200-ms epochs of electrophysiologically recorded swimming activity both under control and strychnine conditions (Fig. 2*C*). We developed a peak detection algorithm (see Materials and Methods) and plotted the probability of peak detection in the 20- to 40-Hz range of the autocorrelation function across age (Peak_20–40_; Fig. 2*D*). At all three developmental stages, the Peak_20–40_ detection scores were reduced by strychnine (Fig. 2*D*, left). However, the attenuation of the Peak_20–40_ detection score by strychnine at 5 dpf was significantly greater than at 3 and 4 dpf (Fig. 2*D*, right), suggesting that the importance of glycinergic neurotransmission to the rhythm driving tail beats changes over time, and a transition occurs between a WGDR to an SGDR around 4–5 dpf in this set of recordings.

**Figure 3.**
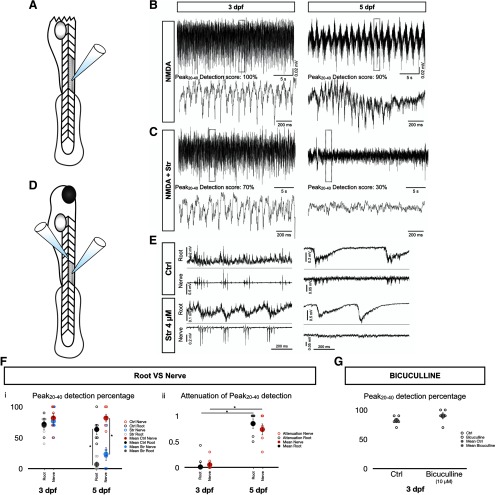
Transition to a SGDR driving tail beats observed in various conditions. ***A***, Schematic of spinalized fish. Fish were cut at the junction between the hindbrain and the spinal cord. ***B***, Upper traces. Typical NMDA induced fictive swimming recording from a 3 dpf (left) and 5 dpf (right) in spinalized fish. Lower traces are magnified views of the dotted boxed regions of the upper traces. ***C***, Same as in ***B*** but after addition of 4 μM strychnine to the bath. ***D***, Schematic of the paired ventral root and motor nerve setup. Muscles overlying somites 4–6 were removed. In this diagram, an electrode was attached to a ventral root from either somite 4 or 5 while another electrode was inserted in the muscle cleft of somite 3 to access the motor nerve. ***E***, Representative traces of paired ventral root and motor nerve recordings under control (up) and strychnine (down) conditions for 3-dpf (left) and 5-dpf (right) zebrafish larvae. ***F***, Peak_20–40_ detection scores (***Fi***) and attenuations of Peak_20–40_ detection (***Fii***) for the ventral root and motor nerve recordings in 3- and 5-dpf fish. Open circles represent the scores of each individual. Solid circles are the averages for every age and recording set-up. *N* = 6 fish; 10 episodes per fish. Error bars represent SEM. For ***Fi***, two-tailed paired Student’s *t* test (3-dpf root Ctrl-Str, *p* = 0.8090; 3-dpf nerve Ctrl-Str, *p* = 0.4149; 5-dpf root Ctrl-Str, *p* = 0.0053; 5-dpf nerve Ctrl-Str, *p* = 0.0009). For ***Fii***, two-tailed paired Student’s *t* test (3-dpf root–3-dpf nerve, *p* = 0.7093; 5-dpf root–5-dpf nerve, *p* = 0.4556) and two-tailed unpaired Student’s *t* test (3-dpf root–5-dpf root, *p* = 0.0007; 3-dpf nerve–5-dpf nerve, *p* = 0.0001); *p < 0.0125, which indicates significance following Bonferroni’s multiple-comparisons correction. ***G***, Peak_20–40_ detection scores under control and bicuculline (10 μM) conditions in 3-dpf fish. Open circles represent the scores of each individual. Solid circles are the averages for every condition. *N* = 6 fish; 10 episodes per fish. Error bars represent SEM. Two-tailed paired Student’s *t* test, *p* = 0.3739.

A number of experimental controls were made to validate our findings. To control for potential contributions from supraspinal centers, we performed recordings on acutely spinalized larvae at 3 and 5 dpf (Fig. 3*A*). Fictive swimming was induced by applying NMDA to the bath (1–1.5 mM). When strychnine was applied, fictive swimming completely stopped in 5-dpf preparations (Fig. 3*C*) but persisted in 3-dpf spinalized preparations (Fig. 3*B*), corroborating our observation of a WGDR to an SGDR transition from 3–5 dpf in the whole animal preparation. In addition, we tested to see whether recording from the ventral root at the exit point from the spinal cord, as opposed to recording from more distal motor nerves as used in some studies (McLean et al., 2008; Tong and McDearmid, 2012; Wiggin et al., 2012), could account for our observation of a WGDR to SGDR transition. This was done in case recordings at the exit point form the spinal cord detected extracellular field potentials from the surrounding neuropil, that could account for the WGDR to SGDR transition. Paired root and motor nerve recordings were made on adjacent somites (somites 3 or 4 based on our numbering system; see Materials and Methods) at 3 and 5 dpf (Fig. 3*D*,*E*). The Peak_20–40_ detection scores of root and nerve recordings at 3 dpf were unaltered by strychnine application (Fig. 3*Fi*). In contrast, strychnine led to an attenuation of the Peak_20–40_ detection scores in the root and in the nerve recordings at 5 dpf (Fig. 3*Fi*). These data indicate that the WGDR to SGDR transition is not a by-product of the proximity of our root recordings to the spinal cord. This is further underscored by the lack of difference in the attenuation of the Peak_20–40_ score due to strychnine between the root and the nerve recordings at either 3 or 5 dpf (Fig. 3*Fii*). In contrast, the attenuations of the Peak_20–40_ score due to strychnine increased between 3 and 5 dpf for both root and nerve recordings, further supporting that a WGDR to SGDR transition is observed for both recording sites. Finally, we examined the contribution of GABAergic transmission to the tail beat rhythm to determine if this inhibitory neurotransmitter was implicated in rhythmogenesis at different stages of development of the larval zebrafish. Application of the GABA antagonist, bicuculline, had no effects on the Peak_20–40_ detection score at 3 or 5 dpf (Fig. 3*G*), suggesting that GABAergic transmission did not contribute to rhythmogenesis of tail beats. Together, these experiments suggest that swimming in developing zebrafish transition from a WGDR to an SGDR between 3 and 5 dpf.

### Differential effect of strychnine along the rostro-caudal axis

**Figure 4.**
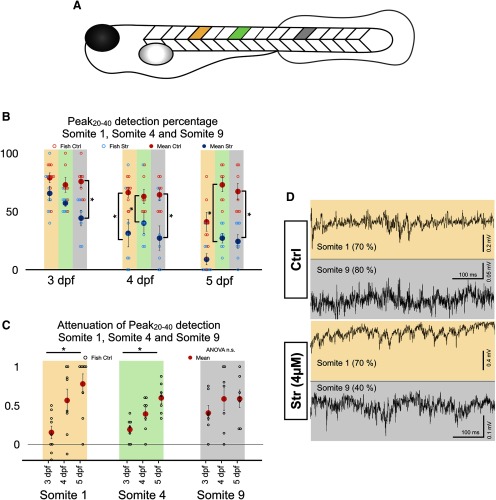
Differential effect of strychnine along the rostro-caudal axis of the zebrafish. ***A***, Schematic of the position at which extracellular recordings were taken: somite 1 (gold), somite 4 (green), and somite 9 (gray). Somites were numbered from 1 to 9 such that somite 1 was the sixth somite rostral to the anus and somite 9 the third one caudal to the anus. ***B***, Results from Peak_20–40_ detection algorithm for autocorrelation functions under control (blue) and strychnine (red) conditions for somites 1, 4, and 9. *N* = 10 episodes per fish in nine fish at every age for somites 1, 4, and 9. Two-tailed paired Student’s *t* test (3-dpf somite 1 Ctrl-Str, *p* = 0.0805; 3-dpf somite 4 Ctrl-Str, *p* = 0.0248; 3-dpf somite 9 Ctrl-Str, *p* = 0.0065; 4-dpf somite 1 Ctrl-Str, *p* = 0.0084; 4-dpf somite 4 Ctrl-Str, *p* = 0.0047; 4-dpf somite 9 Ctrl-Str, *p* = 0.0141; 5-dpf somite 1 Ctrl-Str, *p* = 0.0171; 5-dpf somite 4 Ctrl-Str, *p* = 0.0019; 5-dpf somite 9 Ctrl-Str, *p* = 0.0090). ***C***, Attenuation of Peak_20–40_ detection computed as 1 – (Peak_20–40_Strychnine/Peak_20–40_Ctrl) for somites 1, 4, and 9. One-way ANOVA for each somite (somite 1, *F*_(2,22)_ = 6.286, *p* = 0.0069; somite 4, *F*_(2,18)_ = 7.674, *p* = 0.0039; somite 9, *F*_(2,18)_ = 0.708; *p* = 0.5057) followed by two-tailed unpaired Student’s *t* test (somite 1 3–4 dpf, *p* = 0.0254; somite 1 3–5 dpf, *p* = 0.0017; and somite 1 4–5 dpf, *p* = 0.3772; somite 4 3–4 dpf, *p* = 0.0755; somite 4 3–5 dpf, *p* = 0.0010; and somite 4 4–5 dpf, *p* = 0.093). Top horizontal bars display result of tests between the two groups at each ends of the bars. ***D***, Typical traces of dual recordings from somites 1 and 9 from the same fish (3 dpf) before and after strychnine application. Peak_20–40_ scores for each respective recording in parentheses; *p < 0.0166, indicating significance with Bonferroni’s multiple-comparisons correction. Open circles represent scores of each individual. Solid circles are the averages for every age and condition. Error bars display SEM.

The early development of the zebrafish, including the trunk and the tail, occurs along an anterior-posterior gradient (Kimmel et al., 1995). Since the zebrafish body is still developing between 3 and 5 dpf at the same time that we observed a WGDR to SGDR transition, we asked whether there could also be a gradient in the WGDR to SGDR transition along the length of the spinal cord. To test this, we analyzed recordings at three spinal segments underlying the following somites: the sixth somite rostral to the anus (referred to as somite 1), the third somite rostral to the anus (referred to as somite 4), and the third somite caudal to the anus (referred to as somite 9; Fig. 4*A*). Strychnine decreased the Peak_20–40_ detection score for somites 1, 4, and 9, at 4 dpf and for somites 4 and 9 at 5 dpf (Fig. 4*B*). However, when we compared the effect of strychnine on Peak_20–40_ detection at 3 dpf, strychnine had a significant effect on the caudally located somite 9 but not on somites 1 and 4. This suggests that the caudal somite 9 was already operating by an SGDR at 3 dpf and that somites 1 and four transitioned from a WGDR to an SGDR at later developmental stages. This point is further supported by comparing the attenuation of the Peak_20–40_ detection score by strychnine across the 3- to 5-dpf developmental time window for each somite examined (Fig. 4*C*). The attenuation was significantly increased between 3 and 5 dpf for somites 1 and 4 but not for somite 9. In addition, we performed dual extracellular recordings at 3 and 4 dpf with one electrode situated at the spinal segment underlying somite 1, and another at the spinal segment underlying somite 9, and found examples where strychnine had negligible effects on the 20- to 40-Hz rhythm at somite 1 but had a significant effect on the 20- to 40-Hz rhythm at somite 9 (Fig. 4*D*). No dual extracellular recordings at 5 dpf showed contrasting effects of strychnine on the rhythms recorded at somite 1 versus somite 9. The sum of these experiments suggests that a transition from a WGDR to an SGDR may first occur at caudal domains of the spinal cord where new neurons are integrated into the spinal locomotor circuits to control the most recently developed portions of the tail.

### A subpopulation of sMNs is preferentially generated in the caudal domain of the developing spinal cord at 3 dpf

**Figure 5.**
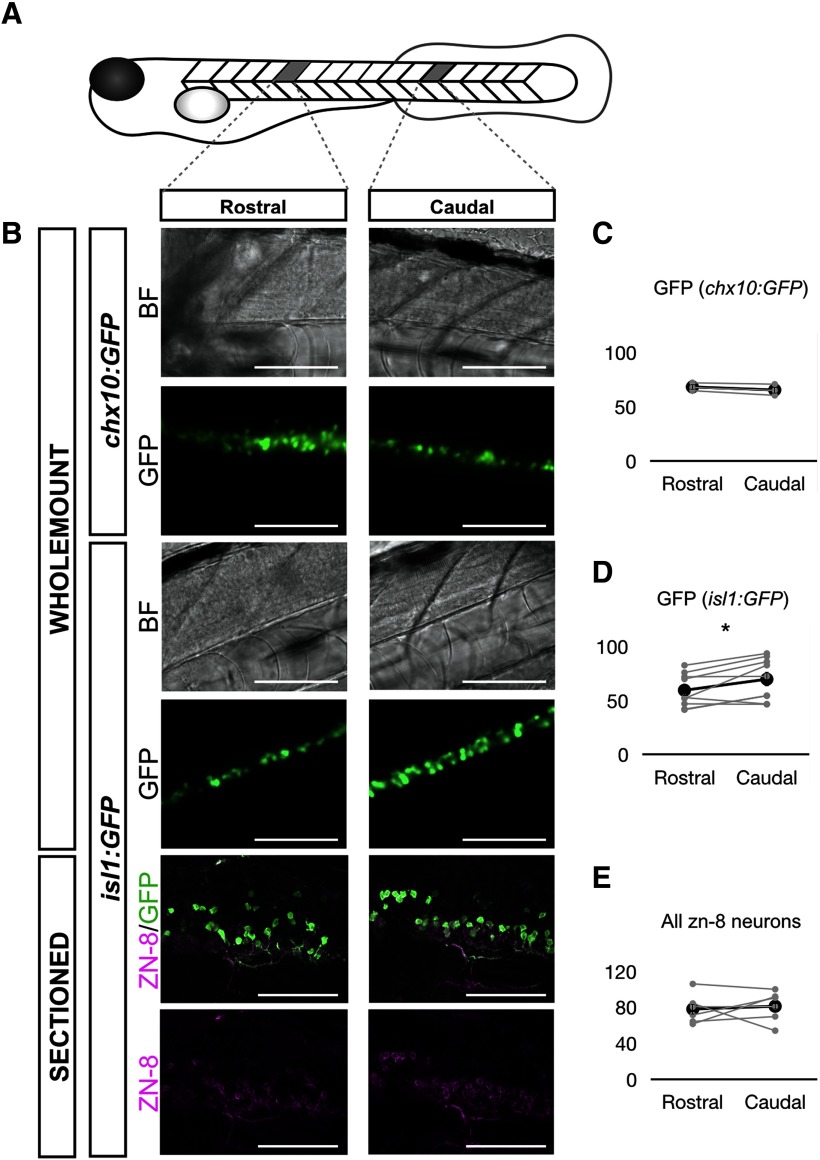
Cell count of sMNs and chx10^+^ spinal neurons in 3-dpf larval zebrafish. ***A***, Schematic of the positions at which Z-stacks were imaged. Counting was performed over two to three somites. ***B***, Representative pictures of targeted rostral and caudal sections, in brightfield (BF) and fluorescent (GFP) conditions for *isl1:GFP* and *chx10:GFP* fish in wholemount preparations, and for *isl1:GFP* fish in sagittal sections of the spinal cord. Scale bars represent 100 μm for wholemount images and 50 μm for section images. ***C***, Count of GFP^+^ cells for the rostral and caudal section in *chx10:GFP* fish. ***D***, Count of GFP^+^ cells for the rostral and caudal section in *isl1:GFP* fish. Counts in wholemount and sectioned fish were pooled together. ***E***, Count of zn-8^+^ cells for the rostral and caudal section in *isl1:GFP* fish. Two-tailed paired Student’s *t* test (GFP^+^ in *chx10:GFP* rostral-caudal, *p* = 0.3196; GFP^+^ in *isl1:GFP* rostral-caudal, *p* = 0.0231; zn-8^+^ in *isl1:GFP* rostral-caudal, *p* = 0.7274); *p < 0.05, indicating significance. Gray circles are the average of three to four independent counts for each fish. Black circles are the average across all fish. Error bars display SD.

To investigate whether the caudo-rostral gradient in the transition from a WGDR to an SGDR is accompanied by variation in the local density of distinct populations of spinal neurons along the rostro-caudal axis, we imaged the expression of a reporter GFP protein along the spinal cord in Tg(*isl1*:*GFP*) and Tg(*chx10*:*GFP*) larvae (Fig. 5*A*,*B*). *isl1* is thought to be expressed in sMNs, whereas *chx10* expression marks V2a (CiD) interneurons that play a central role in rhythmogenesis for locomotion (Ampatzis et al., 2014; Ljunggren et al., 2014; Stil and Drapeau, 2016). sMNs are younger than primary MNs (pMNs) and are primarily involved in swimming as opposed to the latter that are involved in larger, coiling-like movements (Liu and Westerfield, 1988; Ampatzis et al., 2013). Cell counts performed on a rostral location (centered on the seventh somite rostral to the anus) and caudal location (centered on the fourth somite caudal to the anus) showed no differences in V2a interneurons found rostrally (68.2 ± 3.9; *n* = 3 fish) compared with the caudal site (66 ± 6.4; *n* = 3 fish) in Tg(c*hx10*:*GFP*) fish (Fig. 5*C*). Because GFP labeling in the Tg(*isl1:GFP*) line has been reported to be mainly expressed by MNs innervating dorsal axial muscles (Zeller et al., 2002), we repeated immunohistochemical labeling using the antibody zn-8 that has been shown to label a greater proportion of sMNs (Menelaou and Svoboda, 2009). Zn-8 immunostaining revealed more GFP^+^ positive cells in the caudal somites than in the rostral somites (Fig. 5*D*). Nevertheless, there were no differences in the population size of zn-8^+^/GFP^–^ sMNs (rostral: mean ± SD = 29.7 ± 13.5, *n* = 6 fish; caudal: mean ± SD = 28.7 ± 8.3, *n* = 6; *p* = 0.803, two-tailed paired Student’s *t* test) nor the total number of sMNs labeled by zn-8 (Fig. 5*E*). These results imply that the earlier WGDR to SGDR transition occurring in caudal segments of the spinal cord may be associated with greater numbers in caudal segments of a subpopulation of the overall sMN population implicated with slower, more mature forms of swimming.

### Arrhythmic synaptic inhibition to MNs becomes rhythmic after 3 dpf

**Figure 6.**
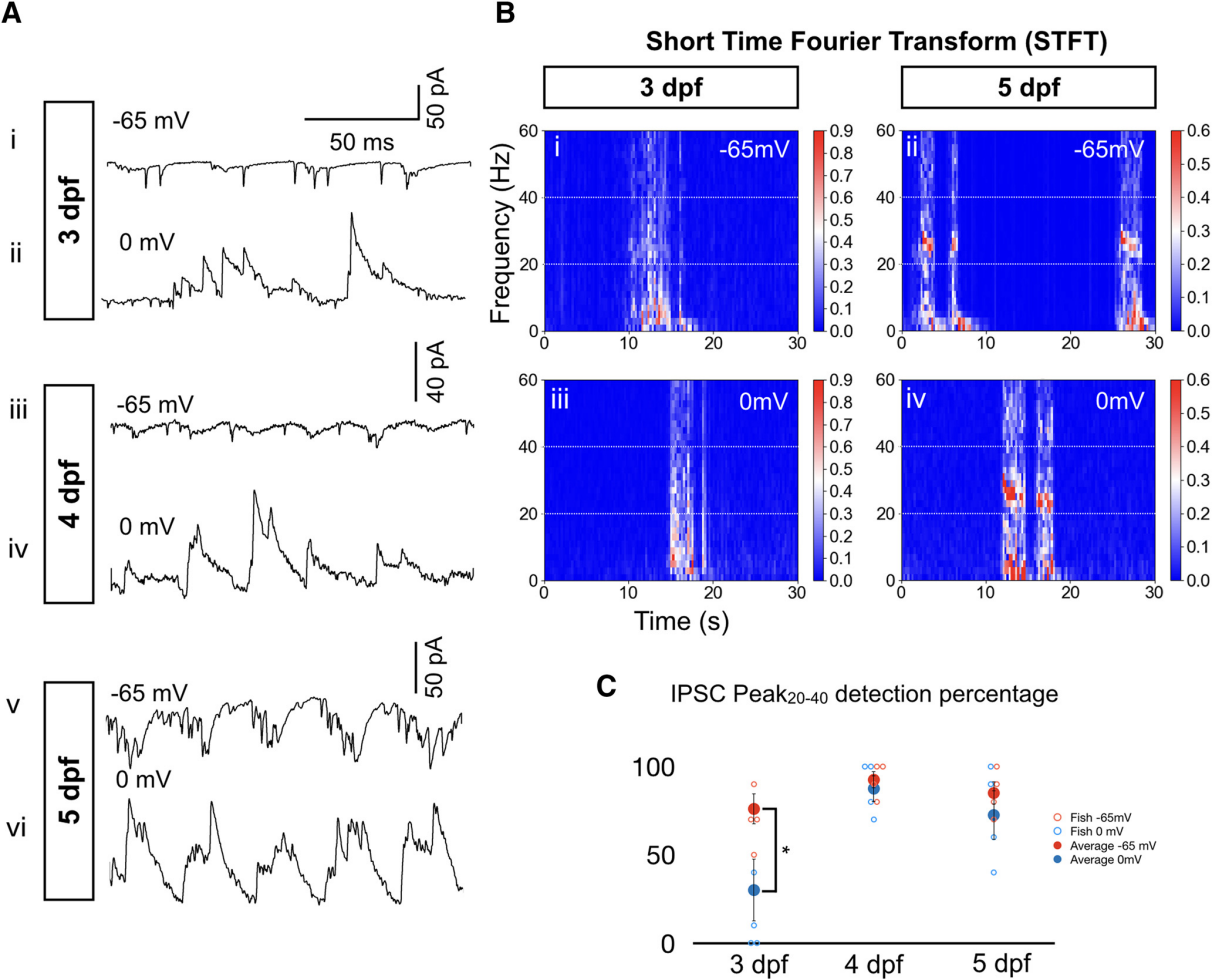
IPSCs mature from arrhythmic at 3 dpf to rhythmic with a frequency close to that of tail beats at 5 dpf during swimming episodes. ***A***, Typical voltage-clamp recordings of MNs during swimming activity at resting (−65 mV) and cation reversal potentials (0 mV) for 3-dpf (***Ai***, ***Aii***), 4-dpf (***Aiii***, ***Aiv***), and 5-dpf (***Av***, ***Avi***) fish. ***B***, Short-time Fourier transform (STFT) of a 30-s-long recording of spinal cord activity under control and strychnine conditions at 3 dpf (***Bi***, ***Bii***) and 5 dpf (***Biii***, ***Biv***). White dotted lines mark the 20- to 40-Hz frequency range. We can observe local maxima in the 20- to 40-Hz range for both 5-dpf plots but not for the 0-mV 3-dpf plot. ***C***, Results from Peak_20–40_ detection algorithm in the 25- to 50-ms time delay range for autocorrelation functions of each traces at 3, 4, and 5 dpf at −65 mV (blue) and 0 mV (red) holding potentials. *N* = 10 episodes per sMN in five sMN at 3 dpf, in four sMN at 4 dpf, and four sMN at 5 dpf. Error bars display SEM. Two-tailed paired Student’s *t* test (3 dpf −65 to 0 mV, *p* = 0.0139; 4 dpf −65 to 0 mV, *p* = 0.3910; 5 dpf −65 to 0 mV, *p* = 0.1942); *p < 0.0166, indicating significance with Bonferroni’s multiple-comparisons correction.

We reasoned that a transition from a WGDR to an SGDR might be reflected through an increase in rhythmicity of glycinergic inputs to MNs (Saint-Amant and Drapeau, 2000; Buss and Drapeau, 2001; Liao and Fetcho, 2008). We proceeded to make whole-cell patch-clamp recordings of secondary sMNs in the spinal cord. In voltage-clamp recordings, we first recorded currents at −65 mV, near resting potential, to assess the rhythmicity of both glutamatergic and glycinergic inputs as the latter have a reversal potential that is depolarized to −65 mV in developing zebrafish (Fig. 6*Ai*,*iii*,*v*). We then recorded currents at the cation reversal potential (0–5 mV; Fig. 6*Aii*,*iv*,*vi*) to isolate outward currents thought to be glycinergic (Buss and Drapeau, 2001). Glycinergic events appeared arrhythmic at 3 dpf (Fig. 6*Ai*), consistent with previous observations (Buss and Drapeau, 2001). However, after this stage, glycinergic events became rhythmic (Fig. 6*Aiii*,*Av*,*B*) as supported by the Peak_20–40_ detection analysis of the autocorrelation functions (Fig. 6*C*). It is interesting to note that all recordings performed between 72 and 78 hpf showed arrhythmic glycinergic events. On the other hand, glycinergic events in MNs that were recorded between the stages of 80 and 84 hpf showed rhythmicity. This switch in the properties of glycinergic currents thus seems to occur within a short time window between the third and fourth day of development and could reflect the WGDR to SGDR switch.

### Chemical ablation of dopaminergic neurons does not preclude a WGDR to SGDR transition

**Figure 7.**
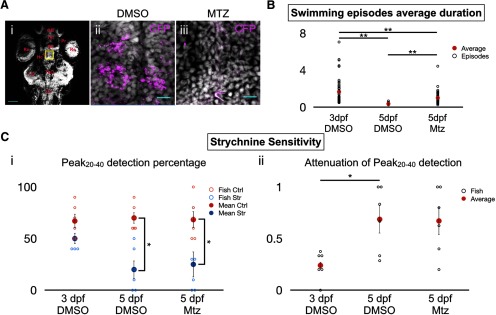
SGDR is preserved in MTZ-treated Tg(*dat:NTR-CFP*) larvae. ***Ai***, DAPI (white)-labeled horizontal section of Tg(*dat:NTR-CFP*) zebrafish brain with high-magnification images of the caudal hypothalamus taken from a (***Aii***) DMSO-treated and (***Aiii***) MTZ-treated fish. OB = olfactory bulbs, Te = telencephalon, Pr = pretectum, DC = diencephalon, Hc = caudal hypothalamus, LC = locus coeruleus, TeO = optic tectum, Ce = cerebellum, Re = retina. Nomenclature as per Godoy et al. (2015). CFP expression is depicted in magenta. Scale bars = 100 μm (***i***) and 10 μm (***ii***). ***B***, Average swimming episode duration (in seconds) calculated for 50 episodes (10 per fish for five fish) at 3 and 5 dpf for DMSO-treated Tg(*dat*:*NTR-CFP*) fish and at 5 dpf only for MTZ-treated Tg(*dat*:*NTR-CFP*) fish. One-way ANOVA (*F*_(2,147)_ = 26.8, *p* = 1 × 10^−10^) followed by two-tailed unpaired Student’s *t* test (3- to 5-dpf DMSO, *p* = 3 × 10^−9^; 3- to 5-dpf MTZ, *p* = 0.0031; and 5- to 5-dpf MTZ, *p* = 2 × 10^−10^); *p < 0.0166, indicating significance with Bonferroni’s multiple-comparisons correction. ***C***, Results from Peak_20–40_ detection algorithm for autocorrelation functions of each extracellular recording at 3 and 5 dpf for DMSO-treated Tg(*dat*:*NTR-CFP*) fish and at 5 dpf for MTZ-treated Tg(*dat*:*NTR-CFP*) fish under control (blue) and strychnine (red) conditions (***Ci***). Attenuation of Peak_20–40_ detection computed as 1 – (Peak_20–40_Strychnine/Peak_20–40_Ctrl) for each fish. *N* = 60 episodes (10 per fish for six fish) for 5-dpf Tg(*dat*:*NTR-CFP*) fish (***Cii***). For ***Ci***, two-tailed paired Student’s *t* test (3-dpf DMSO Ctrl-Str, *p* = 0.019; 5-dpf DMSO Ctrl-Str, *p* = 0.0091; 5-dpf MTZ Ctrl-Str, *p* = 0.0036). For ***Cii***, one-way ANOVA followed (*F*_(2,26)_ = 3.98, *p* = 0.0409) followed by two-tailed unpaired Student’s *t* test (3- to 5-dpf DMSO, *p* = 0.0102; 3-dpf DMSO to 5-dpf NTR, *p* = 0.0398; and 5-dpf WT to 5-dpf NTR, *p* = 0.8155). Top horizontal bars display result of tests between the two groups at each ends of the bars. Error bars represent S.E.M.

Since the WGDR to SGDR transition seems to be concomitant with the transition from burst swimming to beat-and-glide swimming, we asked whether the WGDR to SDGR transition was necessary for the transition in swimming mode. To test this, we sought to prevent the transition from burst swimming to beat-and-glide swimming that normally takes place near 4–5 dpf. Dopamine signaling through D4 receptors during early zebrafish development has been reported to be important in reducing the duration of swimming episodes while increasing the frequency of swim episodes, as seen in the transition from burst to beat-and-glide swimming (Lambert et al., 2012). Therefore we employed a chemogenetic strategy where we chemically ablated nitroreductase (NTR)-expressing dopaminergic neurons of the Tg(*dat*:*CFP-NTR*) transgenic line (Godoy et al., 2015), by application of the pro-drug MTZ (5–7.5 mM; see Materials and Methods). Upon administration of MTZ, NTR is converted into a cytotoxic compound leading to the elimination of a large proportion of dopaminergic neurons (approximately 45–67% decrease according to Godoy et al., 2015). The conditional expression of the fusion protein CFP-NTR by dopamine transporter (*dat*) *cis*-regulatory elements ensured selective ablation of dopaminergic cells when these fish were treated with MTZ (Fig. 7*A*).

Analysis of swimming behavior showed that MTZ application led to longer swimming episodes in 5 dpf Tg(*dat*:*CFP-NTR*) larvae than in DMSO-treated 5 dpf Tg(*dat*:*CFP-NTR*) (Fig. 7*B*), suggesting that ablation of dopaminergic neurons prevented the proper transition from burst swimming to beat-and-glide swimming. This is in agreement with previous reports (Lambert et al., 2012; Godoy et al., 2015). However, swimming episode duration in 5-dpf MTZ-treated fish was shorter than 3-dpf DMSO-treated fish, perhaps due to the incomplete elimination of dopaminergic neurons by MTZ leading to some shorterning of swimming episodes. Nonetheless, we reasoned that if the WGDR to SGDR transition is important for the transition from burst swimming to beat-and-glide swimming, then blocking glycinergic neurotransmission would have weak effects on the swimming rhythm in 5-dpf MTZ-treated fish since these fish still exhibited burst-swimming-like activity. Instead we found that strychnine had a significant effect on rhythm generation in MTZ-treated fish in reducing the Peak_20–40_ detection (Fig. 7*Ci*), and this effect was comparable to 5-dpf DMSO-treated fish (Fig. 7*Cii*). Peak_20–40_ detection in 3-dpf DMSO-treated fish was not affected by strychnine. While there was a difference in the attenuation of Peak_20–40_ detection between 3- and 5-dpf DMSO-treated Tg(*dat*:*CFP-NTR*) fish, there was no difference between 5-dpf DMSO-treated and 5-dpf MTZ-treated Tg(*dat*:*CFP-NTR*) fish (Fig. 7*Cii*). These results suggest that dopaminergic signaling shapes the maturation of the swimming pattern (from long, infrequent swimming episodes to short, frequent episodes) but not the mechanism for rhythm generation within each episode.

### Modelling a change in rhythmogenesis of spinal locomotor networks with two coupled oscillators

**Figure 8.**
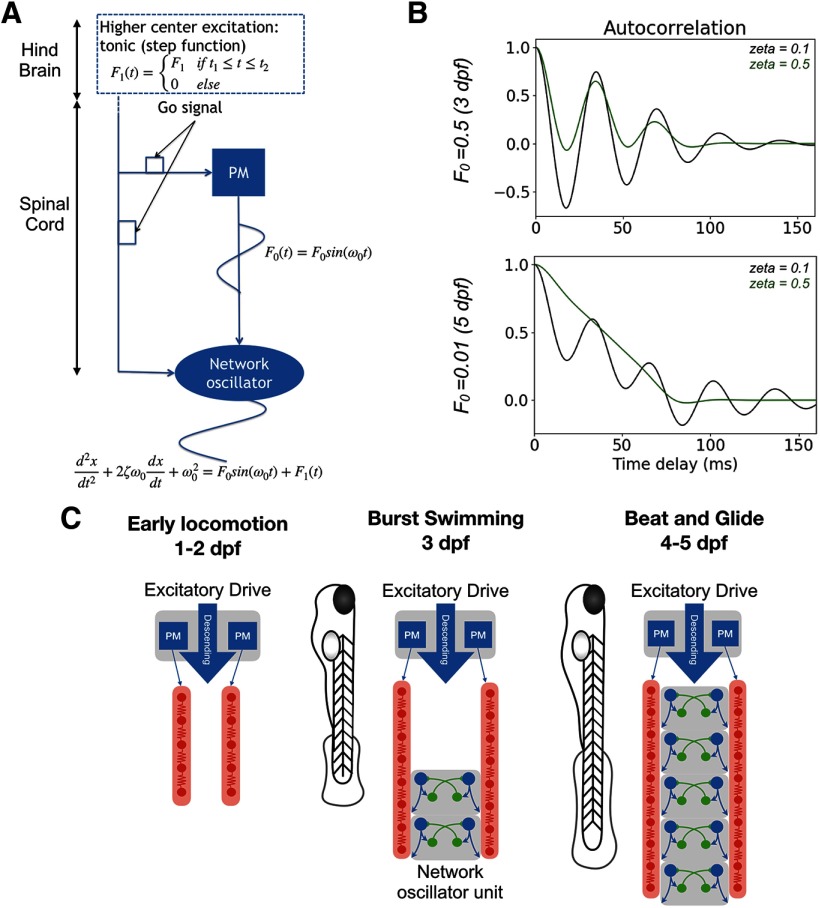
Coupled oscillator model of architectural change from a pacemaker to a network oscillator-based spinal locomotor circuits of developing zebrafish. ***A***, Schematic of coupled oscillators model. Two coupled oscillators: a harmonic oscillator representing a pacemaker (PM) kernel and a damped oscillator representing a half-center network oscillator. The coupling coefficient *F*_0_ represents either the developmental stage or the presence (or absence) of gap-junctions. The damping coefficient models the application or absence of strychnine. The output of the network is read at the output of the half-center. ***B***, Autocorrelation analysis of model output. ***C***, Schematics of the development of spinal locomotor circuits from 3 to 5 dpf. Before 3 dpf, a kernel of IC drives, via gap junctions, two contralateral chains of electrically coupled MNs distributed along the body. Reciprocal inhibition between both sides, which is not illustrated, exists but is not responsible for rhythm generation. After 3 dpf, network oscillators are assembled, first at the caudal end of the spinal cord and then across the length of the spinal cord.

The nascent importance of glycine in rhythmogenesis at later stages of zebrafish development suggests that the control scheme of swimming is changing in the time it takes for burst-swimming to switch to beat-and-glide swimming. Two general mechanisms of rhythmogenesis in neural circuits have been described (Selverston and Moulins, 1985; Harris-Warrick, 2010). The first one is based on pacemaker neurons capable of endogenously bursting when driven by tonic excitation that drives the network rhythm (Wilson and Wachtel, 1974; Tseng and Nadim, 2010). Pacemakers have been proposed to govern locomotor activity in early developing zebrafish through the activity of IC interneurons (Tong and McDearmid, 2012). The second proposed mechanism of rhythmogenesis relies on a network’s connectivity pattern. A classic example of network rhythmogenesis is the theoretical half-center oscillator where reciprocal inhibition of two antagonistic half-centers establishes an alternating activation of the half-centers, thus generating a rhythm (Brown, 1914; Lundberg, 1981). While synaptic inhibition is not a prerequisite to the operation of network-based oscillators, it can be intimately linked to rhythmogenesis as would be the case in a half-center oscillator (Brown, 1914; Lundberg, 1981). An SGDR may be present if swimming at later stages is generated by network oscillators rather than a pacemaker driven network. In other words, before 3 dpf, tail beats may be generated by a pacemaker kernel (represented by PM blue squares in our model; Fig. 8*A*,*C*) that generates swimming episodes due to endogenous bursting properties of constituent neurons such as a persistent sodium current (Tong and McDearmid, 2012). These rhythms are propagated along the length of the fish by a hybrid network involving chemical and electrical synapses (Knogler et al., 2014). In contrast, our data suggest that tail beats at 4–5 dpf are the result of propagating waves generated potentially by network oscillators (represented as a network oscillator unit in the model; Fig. 8*A*,*C*), requiring glycinergic inhibition. The emergence of rhythmic glycinergic inputs in the 20- to 40-Hz frequency range observed in MNs (Fig. 6) supports the notion of developing network oscillators relying on rhythmic synaptic inhibition.

To test the hypothesis that a switch from a pacemaker-driven electrical network to a network oscillator-based spinal locomotor circuit could lead to a WGDR to SGDR transition, we developed a reductive mathematical model. This model consisted of coupled oscillators in which the pacemaker-based rhythmogenic mechanism is modelled by a harmonic oscillator, and the network-based rhythmogenic mechanism (e.g., half-center network oscillator) is modelled by a damped oscillator (Fig. 8*A*). The damping coefficient ζ allows us to model the impact of decreasing synaptic inhibition, such as following the application of strychnine, which was modelled by increasing ζ. The two oscillators have the same natural frequency $\omega_{0}$ of 20 Hz.

Additionally, the two oscillators are coupled by a coupling coefficient $F_{0}$ (see Materials and Methods). We sought to examine whether we could reproduce the WGDR to SGDR transition as resulting from the emergence of a network-based mechanism (i.e., the half-center network oscillator) over a pacemaker-based mechanism. We modelled the reduced influence of the pacemaker-based mechanism as a decrease in the coupling coefficient $F_{0}$. We modelled the application of strychnine at two developmental stages by simulating the network at two different values of ζ (presence or absence of strychnine) and at two different values of $F_{0}$ (high $F_{0}$ representing 3-dpf fish, low $F_{0}$ representing 4–5 dpf). Autocorrelation functions from the network output in our simulations (Fig. 8*B*) reproduced well our experimental data (Fig. 2*C*) under control and strychnine conditions. Therefore, our coupled oscillator model replicated the WGDR to SGDR transition observed experimentally, thus supporting a transition from a pacemaker kernel to network oscillators in spinal locomotor circuits of developing zebrafish.

## Discussion

Zebrafish display movements within hours of fertilization; however, the neural circuits responsible for motor control continue to develop. Developing zebrafish pass through a remarkably rapid transition between sharp, abrupt single coils to more refined rhythmic tail beats within days. Our results reveal that as swimming movements are refined in larval zebrafish, so too are spinal locomotor circuits. We suggest a shift in the role of glycinergic neurotransmission to rhythmogenesis governing tail beats of developing zebrafish in the form of a WGDR to SGDR transition that occurs between 3 and 5 dpf.

In many vertebrate locomotor circuits, glycinergic transmission seems to be dispensable for locomotor activity (Li et al., 2010; although see Moult et al., 2013; Messina et al., 2017). Previous work suggests that this is also the case in the early stages of motor control in the zebrafish (Saint-Amant and Drapeau, 2000, 2001). Our results suggest that glycine remains dispensable for the tail beat rhythm during burst swimming. This is in contrast to what is reported for NMDA-induced swimming in developing zebrafish at 3 and 4 dpf (McDearmid and Drapeau, 2006; Wiggin et al., 2014). This discrepancy may be due to differences in the operation of spinal locomotor networks during NMDA as opposed to during spontaneous or light-induced swimming episodes. The WGDR to SGDR transition was conserved in our experiments with NMDA-induced swimming. The reason a WGDR is observed during NMDA-induced swimming in our study but not in previous studies at 3 and 4 dpf is not clear. We note that a WGDR during NMDA-induced swimming is observed at 3 dpf at lower strychnine concentrations that are close to the ones used in our study (McDearmid and Drapeau, 2006).

We interpret the transition from a WGDR to an SGDR as evidence for a transition from a pacemaker-based operation of spinal locomotor circuits to a network oscillator-based operation. There is strong evidence that pacemakers drive early locomotor movements of the zebrafish. Pharmacologically blocking gap-junctions during the first two days of development arrests locomotor activity in zebrafish, indicating that an early electrical synapse framework is key to the maintenance of locomotor activity at that stage (Buss and Drapeau, 2001). Recently, the rostral spinal IC neurons have been identified as putative pacemakers for swimming at this developmental stage due to their endogenous bursting driven by persistent sodium currents and their electrical connections to MNs (Tong and McDearmid, 2012).

As opposed to pacemakers that can intrinsically generate oscillatory activity, network oscillators rely on synaptic transmission for rhythmogenesis. Depending on the structure of a network oscillator, synaptic inhibition can be critical for the generation of a rhythm, as illustrated in the theoretical half-center oscillator (Brown, 1914; Lundberg, 1981). While glycinergic currents have the particularity of being inward (i.e., depolarizing) at resting potential in developing zebrafish (Saint-Amant and Drapeau, 2000, 2001), the proximity of its reversal potential to the resting potential would lead glycinergic neurotransmission to act as a shunting inhibition (Ben-Ari, 2002; Drapeau et al., 2002; Knogler and Drapeu, 2014). Therefore, we suggest that glycinergic inhibition at early developmental stages is still a viable means of implementing reciprocal inhibition between half-centers in the developing zebrafish and the WGDR to SGDR transition is indicative of a transition away from a pacemaker to a network oscillator mechanism of rhythmogenesis. While we did not assess whether the glycinergic reciprocal inhibition between half-centers in developing zebrafish is contralateral or ipsilateral, both types of synaptic inhibition could play separate roles in coordinating left-right alternation and rhythmogenesis (Gabriel et al., 2008). Evidence from the adult lamprey suggests that contralateral inhibition could play a role in maintaining a swimming rhythm (Messina et al., 2017). Recent studies identified V1 interneurons marked by engrailed-1 expression and V2b interneurons defined by expression of Lhx3 and Gata3 as ipsilaterally projecting inhibitory interneurons that play contrasting roles in locomotor control. V1 interneurons seem to shorten the duration of swimming episodes and derecruit slow-muscle innervating MNs (Kimura and Higashijima, 2019), whereas V2b interneurons provide a break to locomotor speed (Callahan et al., 2019). How the combined activity of these populations shapes the rhythm of tail beats during swimming episodes, and whether their contributions change as fish transition from burst swimming to beat-and-glide swimming remains to be investigated.

Surprisingly, we found that the WGDR to SGDR transition did not occur uniformly across the length of the spinal cord but instead occurred in a caudo-rostral direction. The direction of the WGDR to SGDR transition appears to run counter to the rostro-caudal direction of growth of the developing zebrafish where more caudal somites are much smaller earlier in development than their rostral counterparts (Kimmel et al., 1995). Anatomically, our cell counts of sMNs reveal a greater number of sMNs innervating dorsal axial muscles in caudal segments at the studied ages, with the caveat that part of this GFP^+^ population could be a population of spinal interneurons ectopically expressing GFP at 3 dpf. Nevertheless, considering that pMNs and sMNs are primarily dedicated to swimming movements of different speeds (Liu and Westerfield, 1988), it is possible that nascent swimming networks at the caudal end of the growing fish first establish spinal circuits dedicated to controlling sMNs as opposed to pMNs, with these newer circuits reliant on synaptic inhibition in rhythmogenesis. We thus propose that the emerging importance of glycinergic transmission to the rhythm of tail beats is due to the establishment of network oscillators within spinal locomotor circuits of the zebrafish that are first established in caudal segments of the developing zebrafish spinal cord. Network oscillators are then subsequently established in progressively more rostral segments as the zebrafish continues to develop.

We used computer simulations to test whether an increased reliance on glycinergic transmission could arise from a decreasing drive from pacemakers to spinal locomotor circuits. Our simulations involved a pair of coupled oscillators, one representing a pacemaker-mechanism of rhythmogenesis, the other representing network oscillators involving synaptic inhibition for rhythmogenesis. These simulations supported the notion that a relatively decreasing drive from pacemakers to spinal locomotor circuits during development, putatively due to the addition of new spinal circuitry, could lead to locomotor activity that relies more strongly on glycinergic transmission. We modelled spinal locomotor circuits as a series of longitudinally distributed network oscillators, as opposed to a single oscillator, in part because of our finding that the WGDR to SGDR transition does not occur uniformly across the length of the spinal cord. Multiple longitudinally distributed network oscillators in the zebrafish spinal cord are also supported by other experimental studies where very short 5 dpf spinal cord segments can generate rhythmic activity (McDearmid and Drapeau, 2006; Wiggin et al., 2012).

Over time, there is a possibility that two overlapping networks arise. The first is an electrical network linking pacemaker neurons and pMNs used for larger movements such as coils and perhaps escape responses. The second includes a network based on longitudinally distributed half-center network oscillators linking sMNs to neural circuits dedicated to slower forms of swimming. It is however likely that both electrical and chemical synapses are concomitantly involved during some swimming activity, as suggested by the presence of both types of synapses in the V2a-MN circuit (Song et al., 2016). Similarly, a hybrid pacemaker-network oscillator has been proposed to underlie different speeds of locomotion in mammalian locomotor circuits (Brocard et al., 2010).

### Role of dopaminergic neurons in pattern generation

The WGDR to SGDR transition becomes important to the rhythm of tail beats during the transition from burst swimming to beat-and-glide swimming patterns. However, the coincidence of these two transitions, one governing the rhythm of tail beats, the other setting the pattern of these tail beats, does not mean that they are causally related. Previous studies have suggested that dopamine through D4 receptor signaling is essential for the maturation to beat-and-glide swimming (Lambert et al., 2012). While our observed phenotype was closer to burst swimming at 5 dpf when dopaminergic neurons were partially ablated, strychnine effectively disrupted the tail beat rhythm. While we cannot definitively conclude that the transition from a WGDR to an SGDR is completely disconnected from the swimming phenotype transition from burst to beat-and-glide swimming, other mechanisms would be required to explain this maturation such as perhaps the modulation of an ultraslow hyperpolarization (Zhang et al., 2015).

### Future directions

Our findings could relate to the development of motor circuits in other vertebrates. For instance, it seems that an early electrical scaffold is necessary for the development of mice spinal locomotor circuits (Tresch and Kiehn, 2000). Whether an electrical network acts as a monolithic block from which locomotor circuits will later be sculpted and refined via new chemical connections to accommodate more precisely defined locomotor movement needs to be determined across vertebrates. Our results suggest that the circuits involved in rhythmogenesis operate as network oscillators, an important finding considering the continued failure to identify pacemakers solely responsible for driving locomotor activity in many well-characterized vertebrate spinal locomotor circuits (Ziskind-Conhaim and Hochman, 2017).

In closing, it is interesting to note that knockdown of glycinergic receptors leads to reduced numbers of spinal interneurons and increased numbers of mitotic cells in the developing zebrafish spinal cord (McDearmid et al., 2006; Côté and Drapeau, 2012). This opens up the intriguing possibility that the role of glycine in the maturation of spinal locomotor circuits is not restricted to its emerging mechanistic contribution to rhythmogenesis but perhaps to the development of populations that make up newly formed spinal locomotor circuits.

## Synthesis

Reviewing Editor: Muriel Thoby-Brisson, CNRS UMR 5287 Université Bordeaux Decisions are customarily a result of the Reviewing Editor and the peer reviewers coming together and discussing their recommendations until a consensus is reached. When revisions are invited, a fact-based synthesis statement explaining their decision and outlining what is needed to prepare a revision will be listed below. The following reviewer(s) agreed to reveal their identity: Mark Masino, Abdeljabbar El Manira. Note: If this manuscript was transferred from JNeurosci and a decision was made to accept the manuscript without peer review, a brief statement to this effect will instead be what is listed below.

This study, although based on a sound scientific premise, suffers from poor experimental design, potentially faulty statistics, and weak, and often incorrect, interpretation of results. The manuscript is not written well and includes jargon, incorrect spelling, colloquialisms, and poorly constructed sentences. The Methods are not sufficiently described.

Major Comments:

• Lines 78-80: The authors’ statement that “McDearmid and Drapeau (2006) showed that glycinergic neurotransmission... is dispensable for rhythm at 3 dpf” is incorrect. This statement is contradicted by the results presented by McDearmid and Drapeau. First, high concentration of Strychnine (10 uM) produced co-incident multiple waves along the rostrocaudal axis, which indicated a loss of coordination. Second, the locomotor activity was differentially affected by the concentration of Strychnine. At high levels (12 uM), locomotor activity was abolished. WHY? At low concentrations (6 uM), the locomotor activity was synchronous from side-to-side. None of these results are consistent with the authors’ statement. In fact, these results argue the opposite, that glycinergic neurotransmission is INDISPENSABLE.

• The authors should correct the misleading statement regarding sex/gender of the larvae. This is not ‘unknown’, rather it is developmentally undetermined at this stage of development.

• The authors are strongly encouraged to have this manuscript edited by an individual with knowledge of correct grammar. There are instances, too many to identify, of incorrect spelling, use of prepositions, use of jargon and/or colloquialisms, and verb tense throughout the manuscript.

• Line 153: Explain the rational for using 2M NaCl solution in the extracellular recording electrodes. What is the relevant reference used as a precedent?

• Line 172: How were the motoneurons targeted? Was a transgenic line used? Were MNs backfilled? Was it based on dorsoventral position in the spinal cord.

• Line 172: How were the identities of recorded MNs confirmed?

• Lines 231-36: How often were animals rejected?

• Line 284: The authors claim that they intend ‘To identify the rhythmogenic mechanism underlying tail beats’ using high-speed video recordings is incorrect. These data will not provide insight into the mechanism of rhythmic activity. Rather, it permits them to assess the kinematics of rhythmic activity; there is not an investigation of the mechanisms.

• Lines 291-296: See comments above for Lines 78-80. Of serious concern is that the authors claim that strychnine blocks left-right alternation without affecting the rhythm of the tail beats here, but earlier (Lines 78-80) states that ‘glycinergic neurotransmission... is dispensable for rhythm’.

• Lines 301-04: Please state explicitly what the control traces represent (i.e. extracellular peripheral nerve or ventral root recordings).

• Lines 321-22: The concentration of NMDA should be indicated in the Methods section. Also, 1-1.5 mM NMDA is an incredibly high concentration. Most papers use on the order of 100-250 uM to produce NMDA-induces fictive-locomotion. Concentrations at and above 200 uM tend to eliminate the motor activity within 10s of minutes, likely due to excitotoxicity. These data are suspect if the concentrations indicated are correct.

• Lines 324-27: What is the rationale and/or basis for examining the difference between recordings of the ventral root at proximal and distal sites? This logic is not well developed and does not further the understanding of the work.

• Lines 331-333: This sentence is not well written and difficult to follow. An editor can help convey the information.

• Lines 335-36: It is likely that the statistics, based on the graph in figure 3Fii, were done incorrectly. The attenuation of the peak detection in the root appears to be statistically different between control and strychnine application.

• Lines 342-43: The authors fail to indicate why they decided to investigate GABA synapses.

• Lines 343-45: Were statistics performed for experiments shown in figure 3G? If so, state the results and remove the unnecessary term ‘negligible’.

• Lines 390-91 and 394-97: The interpretation of greater MNs expressing GFP in more caudal segments (figure 5D) is probably due to misexpression of GFP in non-MN cells. First, the Tg(isl1:GFP) line is not limited to MNs; there is ectopic expression in other spinal neurons. This is an important point. Many labs use transgenic lines that are supposed to mark MNs, but the labs do not evaluate are know whether the expression is specific or not. In this case, the GFP expression is not specific to MNs and is likely expressed in more non-MNs at earlier developmental stages. Second, GFP is likely expressed in more non-MNs in more caudal segments due to the rostrocaudal progression of development. Finally, what is the evidence for the statement that there is greater neurogenesis in the caudal segments? Therefore, the data presented does not convince this reviewer that more MNs are present at more caudal segments.

• Lines 435-41: Of course blocking glycinergic inhibition with strychnine produces an effect on the locomotor rhythm. The authors’ hypothesis that blocking glycinergic inhibition in 5 dpf MTZ-treated fish should have a weak effect on the rhythm is baseless. Glycinergic inhibition is required for left-right coordination of motor activity; it has not been shown to perturb the pattern (i.e. duration of locomotor activity). Lambert et al. (2012) showed that the duration of swimming was due to D4R receptor signaling, not strychnine and did not claim any effect on rhythm generation. The authors should read this paper again.

• Lines 451-56: This sentence makes no sense. No data presented to evaluate the claim.

• Lines 571-73: I have not been convinced that they have confirmed a role of glycinergic neurotransmission due to a weak to strong glycine-dependent rhythm.

• Lines 693-94: Expand on and explain why/how glycine is important for neurogenesis and cell-fate. This sentence is hollow with further context.

• Figure 1A & B: Why are the locomotor outputs in A and B different? Clearly, the raw video frames (A) are not fully represented in the extracted midlines (B).

• Line 814 and Figure 2A: These are not ‘typical extracellular recordings of fictive swimming activity’ as stated. First, extracellular recording typically have a stable baseline; the traces shown here are not stable. There is not sufficient information provided in the Methods to permit the reader to understand this difference, and so confidence is reduced. I am concerned that this is actually 50-60 cycle noise that was not eliminated from the recording.

• Figure 7 and legend: Nothing stated regarding CFP. Are the ‘magenta’ indicated cells supposed to be DAPI or CFP? This is not explicitly stated in the text.

• Figure 7D: How are the data for 3 and 5 dpf presented in this panel different than those presented in Figure 6C? Importantly, they are not different and the authors are using the same data across multiple figures.

• Figure 7B & D: These experiments are poorly designed. The data should have been collected from non-transgenic siblings at 3 and 5 dpf, rather than from WT as stated.

Minor Comments:

• Title: The title is too vague as it relates to the switch (switch in what?) and would benefit from an explicit statement regarding a switch in glycine-mediated synaptic input. However, since there are significant Major Comments that reduce the enthusiasm of this manuscript, a title change will not help the overall review.

• References: The date of publication for the McDearmid and Drapeau reference is incorrect.

• Line 132: How does one perform a skin flap? The authors intend to state that a skin flap was made and then used to remove the skin of the larva.

• Please provide complete information regarding the borosilicate glass used, including i.d. and o.d. dimensions and the model/part number from A-M Systems.

• Line 163: Indicate the concentrations of strychnine and bicuculline.

• Line 183: 3dpf larvae not indicated in figure legend.

• Line 213: State the dilution of goat anti-mouse secondary antibody.

• Line 303: Poor use of phrasing (‘easily noticeable’).

• Line 346: State explicitly how many extracellular recordings were performed.

• Line 428: State the concentration of MTZ used in this experiment.

• Lines 449-50: IPSPs were not recorded; IPSCs were.

• Lines 504 and 506: The references for ‘Pacemaker’ and ‘Network’ rhythms are fine, but there are more appropriate and seminal one that should have been cited.

• Line 515: What is ‘later swimming’?

• Line 518: Endogenous bursting properties of what? State explicitly.

• Lines 542-45: Sentence is poorly constructed to basically state that they simulated by simulating.

• Lines 661-64: This sentence does not make sense and needs to be rewritten for clarity.

• Line 678: Replace ‘definitely’ with ‘definitively’.

• Lines 694-98: Use of the term ‘emerging’ twice in the same sentence.

• Figure 1C and legend: The dotted black lines are not defined.

• Figure 1D and legend: The dotted white lines are not at 20 and 40 Hz as indicated. They are close, but not at those values in the figure.

• Figure 3E: Again, why is the baseline changing in these ‘extracellular’ recordings?

• Figure 3Fi: It does not make sense that the peak detection percentage at 5 dpf is different for the ‘Nerve’ but not the ‘Root’. Based on the results presented in the figure, I suspect the statistics were not performed accurately.

• Figure 5 and legend and Methods: Indicate what ages were used for cell counts.

• Figure 7B: Indicate the units (sec) for the Y-axis.
